# Strut Size-Dependent Compressive Behavior and Failure Mechanisms of Laser-Based Powder Bed Fusion NiTi Octahedral Porous Scaffolds

**DOI:** 10.3390/ma19050951

**Published:** 2026-02-28

**Authors:** Ning Zhang, Wangwei Zhan, Hongsen Liu, Chuanhui Huang, Guangqing Zhang, Yinghong Zhang, Jinguo Ge

**Affiliations:** 1 School of Mechanical and Electrical Engineering, Xuzhou University of Technology, Xuzhou 221018, China; 2 School of Mechanical and Electrical Engineering, Guilin University of Electronic Technology, Guilin 541010, China; 3 Department of Mechanical and Manufacturing Engineering, Trinity College Dublin, The University of Dublin, Parsons Building, D02 PN40 Dublin, Ireland

**Keywords:** selective laser melting, NiTi alloy, porous scaffold, finite element simulation, compression behavior

## Abstract

Nickel-titanium (NiTi) alloys are attractive for functional and biomedical applications due to their shape memory effect, superelasticity, and favorable corrosion resistance and biocompatibility. In this work, the influence of strut size on the compressive response of laser-based powder bed fusion (PBF-LB/M) fabricated NiTi ortho-octahedral porous scaffolds was systematically investigated using combined experiments and finite element simulations. Four scaffold designs with identical unit-cell size (2 mm) but different strut sizes (280, 320, 360, and 400 μm) were fabricated, and their forming quality and deformation behaviors were examined. The as-built scaffolds exhibited high geometric fidelity to the CAD models and stable manufacturability across the investigated parameter range. Quasi-static compression tests revealed a typical three-stage response (linear-elastic regime, plateau/collapse regime, and densification), with both elastic modulus and compressive strength increasing markedly with strut size. Specifically, the modulus increased from 1.17 to 4.28 GPa and the compressive strength increased from 155 to 564 MPa as the strut size increased from 280 to 400 μm. A pronounced oscillatory plateau was observed for the 280 μm scaffolds, indicating progressive layer-by-layer collapse, whereas larger struts promoted a shear-band-dominated failure mode characterized by an approximately 45° fracture zone. Explicit quasi-static simulations reproduced the experimentally observed collapse sequence and demonstrated that stress preferentially concentrates at nodal junctions, with load transfer dominated by struts aligned with the loading direction. The agreement between experiments and simulations confirms the predictive capability of the proposed modeling framework and provides mechanistic insights into geometry-controlled failure. These findings establish a structure-property-failure relationship for PBF-LB/M-fabricated NiTi octahedral scaffolds and offer practical guidance for tailoring stiffness, strength, and collapse mode through strut-size design.

## 1. Introduction

Near-equiatomic nickel-titanium (NiTi) alloys have attracted sustained interest as advanced functional materials owing to their pronounced shape memory effect and superelasticity, together with excellent corrosion resistance and biocompatibility [1]. These attributes have enabled widespread applications in aerospace, automotive, and biomedical engineering. In particular, NiTi is regarded as a promising candidate for load-bearing orthopedic implants because its elastic modulus (typically 28–75 GPa, depending on composition and processing history) is closer to that of natural bone than many conventional implant metals, thereby helping mitigate stress shielding and improving mechanical compatibility with host tissues [2,3]. Beyond the intrinsic merits of the alloy, introducing designed porosity is a powerful strategy to further tailor stiffness and enhance biological fixation. Architected porous scaffolds can provide low density and high specific mechanical performance, while also offering interconnected pores and a large surface area to facilitate cell infiltration, proliferation, and differentiation, features that are beneficial for osseointegration and long-term implant stability [4,5]. Similar structural advantages also motivate the use of porous lattices in lightweight engineering applications where energy absorption and weight reduction are desired.

Despite these advantages, manufacturing NiTi components, especially with controlled porous architectures, remains challenging using conventional processing routes. NiTi alloys are highly sensitive to impurity pickup and oxidation because of their high Ti activity, and high-temperature melting/casting can introduce compositional fluctuations and defects that compromise functional and mechanical performance [6]. Moreover, the combined characteristics of ductility, elasticity, adhesion, work hardening, and springback complicate post-processing and machining, leading to severe tool wear and degraded surface quality [7]. Additive manufacturing (AM), particularly selective laser melting (PBF-LB/M), provides an attractive alternative because it enables near-net-shape fabrication directly from CAD models, reduces material waste, and offers the capability to produce complex lattice geometries with high dimensional controllability [8,9,10,11]. Accordingly, PBF-LB/M has been extensively explored for metallic lattices and porous components made from Ti alloys, stainless steels, aluminum alloys, and high-entropy alloys, where the relationships among process-structure-property have been progressively clarified [10,12,13,14]. For porous scaffolds, Ti-6Al-4V remains the dominant material system in the literature [15,16]; however, with the rapid maturation of PBF-LB/M equipment and parameter optimization, NiTi porous architectures are increasingly being recognized for their unique combination of functional behavior and mechanical compatibility [17,18,19].

For porous lattices fabricated by PBF-LB/M, mechanical performance is governed not only by the base material but also, often more critically, by structural parameters such as strut diameter, unit-cell topology/size, and overall porosity/relative density [20,21,22]. Prior studies on TPMS-based gradients and BCC-type lattices, largely focusing on Ti alloys, have demonstrated that geometric tailoring can markedly alter stiffness, strength, and energy absorption, and that stress concentrations frequently develop at junction regions where struts meet [23,24,25]. These findings collectively imply that, even for a fixed unit-cell topology, relatively subtle variations in strut dimensions may trigger substantial changes in deformation pathways and failure modes. However, compared with the extensive body of work on Ti lattices, systematic investigations on NiTi porous scaffolds remain limited, especially those linking strut size to compressive response and failure mechanisms through combined experiments and validated simulations. This knowledge gap is nontrivial: NiTi’s distinct constitutive behavior and defect sensitivity under PBF-LB/M processing may lead to deformation and fracture characteristics that cannot be directly extrapolated from titanium-based scaffolds. In addition, for clinical and engineering implementation, it is not sufficient to report only peak strength or elastic modulus; it is also essential to understand where damage initiates (e.g., nodal junctions vs. strut mid-spans), how collapse progresses (progressive layer-wise crushing vs. shear-band dominated collapse), and why different architectures exhibit different stress–strain signatures (e.g., oscillatory plateau vs. smoother hardening), all of which directly determine reliability, energy absorption, and safe design windows.

To address these issues, the present study employs PBF-LB/M to fabricate four sets of NiTi porous scaffolds based on an ortho-octahedral (octahedral) unit-cell topology while systematically varying strut size. This topology is frequently considered advantageous for interconnected pore networks and structural connectivity, which are desirable for scaffold applications. By coupling quasi-static compression experiments with finite element simulations, this work aims to (i) quantify how strut dimension influences elastic modulus and compressive strength under identical unit-cell size, (ii) reveal the dominant deformation and failure modes and their transitions with increasing strut size, and (iii) determine the stress localization features within unit cells (especially at nodal junctions) to rationalize the experimentally observed collapse patterns. Importantly, the simulation framework is designed to be validated against experimental behavior, thereby providing a predictive tool to interpret failure mechanisms and guide subsequent scaffold design. Through this combined experimental-numerical approach, the study establishes a clearer structure-property-failure relationship for PBF-LB/M-fabricated NiTi porous scaffolds, offering design-relevant insights for both biomedical and lightweight structural applications.

## 2. Test Method and Finite Element Simulation

### 2.1. Positive Octahedral Porous Scaffold Design

Previous studies have demonstrated that the ortho-octahedral lattice is particularly favorable for applications involving bone tissue regeneration and load-bearing biomedical implants. Lewis Mullen et al. [26] systematically investigated the structural characteristics of ortho-octahedral units and showed that this topology provides an advantageous balance between mechanical integrity and biological functionality. By analyzing parameters such as pore size, porosity, and structural connectivity, they found that the ortho-octahedron exhibits the smallest spatial residual volume among commonly used ortho-polyhedral lattices, together with a high degree of nodal connectivity. These features enable efficient load transfer while minimizing unnecessary material usage, which is critical for lightweight scaffold design. In addition, the interconnected pore network inherent to the ortho-octahedral geometry forms continuous through-holes that facilitate nutrient transport, waste removal, and cell migration, thereby promoting bone tissue ingrowth and maturation [27].

**Figure 1:**
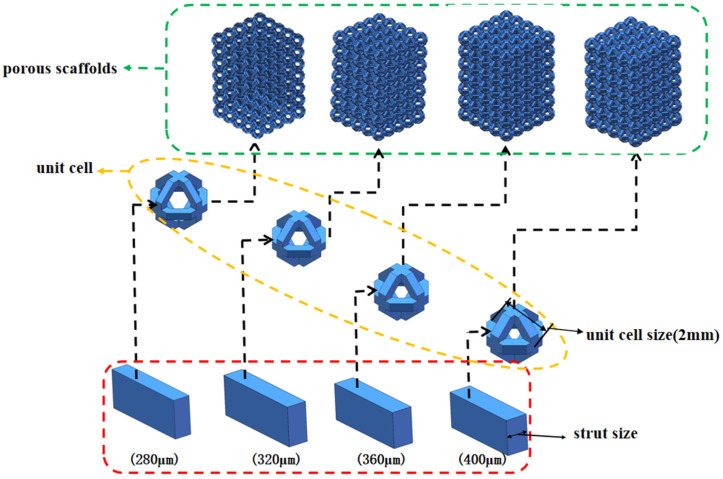
Schematic illustration of the ortho-octahedral unit cell and the assembled porous scaffold architecture with different strut sizes.

Based on these considerations, the ortho-octahedral topology was selected as the fundamental unit-cell architecture in the present study. Within each individual unit cell, the inclined struts form a regular octagonal configuration, in which the adjacent trusses intersect at an angle of 135°, as illustrated in Figure 1. Four types of NiTi ortho-octahedral porous scaffolds were designed by systematically varying the strut size while keeping the unit-cell size constant. The cell size was fixed at 2 mm to isolate the effect of strut dimension on mechanical response. The corresponding strut diameters were 280 μm, 320 μm, 360 μm, and 400 μm, yielding relative densities of 15.13%, 18.88%, 22.88%, and 27.00%, respectively, as summarized in Table 1. As the strut size increased, the minimum cross-sectional area perpendicular to the loading direction also increased, with values of 9.52 mm^2^, 10.56 mm^2^, 11.52 mm^2^, and 12.40 mm^2^, respectively. This systematic variation establishes a clear geometric basis for analyzing the influence of strut dimension and effective load-bearing area on the compressive behavior discussed later.

The porous scaffolds were constructed by arranging the ortho-octahedral unit cells in a periodic manner, with 5 × 5 × 7 cells along the X-, Y-, and Z-directions, respectively. This configuration resulted in overall specimen dimensions of 10 × 10 × 14 mm, ensuring sufficient structural height to capture progressive deformation and failure modes under compression. All unit cells and scaffold assemblies were modeled using SolidWorks (SolidWorks 2022, Dassault Systèmes, Vélizy-Villacoublay, France). Individual octahedral cells were first generated and then replicated through mirroring and array operations to form the complete lattice structure. By maintaining identical external dimensions and unit-cell size while varying only the strut diameter, four distinct scaffold designs with well-controlled geometric parameters were obtained, as illustrated in Figure 1. This design strategy allows the subsequent experimental and numerical analyses to directly correlate differences in compressive response, stress distribution, and failure mechanisms with strut size and relative density, rather than with changes in topology or specimen scale.

### 2.2. Preparation of Compression Specimens

**Figure 2:**
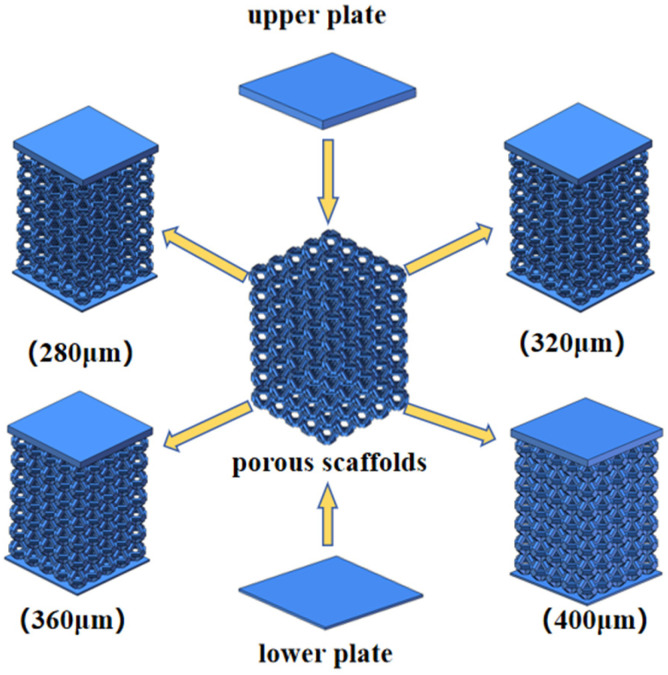
Geometric configuration of the ortho-octahedral porous scaffold with integrated upper and lower cover plates for compression testing.

The compression specimens were designed based on the previously described ortho-octahedral porous scaffold with overall dimensions of 10 × 10 × 14 mm. To ensure stable and repeatable boundary conditions during compression testing, rigid upper and lower cover plates were incorporated into the scaffold design, forming a porous structure with integrated end caps, as illustrated in Figure 2. The final geometry consisted of a 0.76 mm-thick upper plate, a porous intermediate section, and a 0.2 mm-thick lower plate, creating a composite configuration suitable for uniaxial compression. The inclusion of cover plates serves two primary purposes: first, to provide a uniform load transfer from the compression platens into the porous architecture; and second, to reduce premature local failure at the specimen-platen interface, thereby allowing the intrinsic deformation and collapse behavior of the lattice to be more clearly observed.

**Figure 3:**
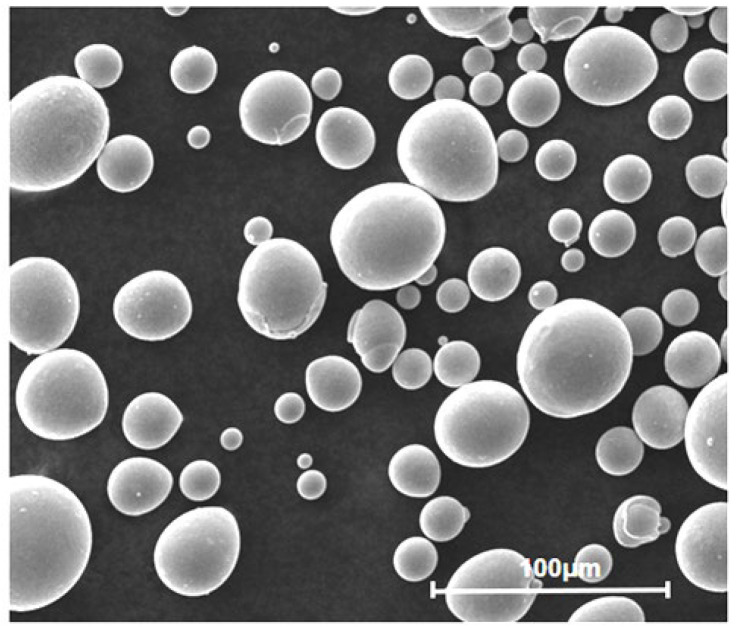
SEM images of the near-equiatomic NiTi powder used for the present work.

Near-equiatomic NiTi powder was employed as the raw material and was supplied by Jiangsu Vilory Advanced Materials Technology Co. (Xuzhou, China), ensuring adequate compositional control for the fabrication of functional porous scaffolds. Figure 3 presents representative scanning electron microscope (SEM, Quanta 450 FEG, FEI Company, Hillsboro, OR, USA) images of the powder, illustrating its morphology and size distribution. The powder particles exhibited a size range of 25–53 μm, with a nominal composition of 49.57 at.% Ti and 50.43 at.% Ni. Such a particle size distribution is suitable for stable powder spreading and consistent melting behavior during the PBF-LB/M process.

All specimens were fabricated using an EOS M290 selective laser melting system (EOS GmbH, Krailling, Germany). The processing parameters were optimized based on prior studies and preliminary trials [28], and were fixed for all specimens to isolate the effect of geometric parameters on mechanical performance. The PBF-LB/M parameters included a laser power of 200 W, a scanning speed of 1100 mm/s, a hatch spacing of 0.1 mm, and a layer thickness of 30 μm. To minimize oxidation and compositional deviation during fabrication, high-purity argon gas was continuously supplied to the build chamber, maintaining an inert processing atmosphere throughout the manufacturing process. A layer-wise rotated scanning strategy was employed during PBF-LB/M fabrication. The laser scan direction between adjacent layers was rotated by 67°, i.e., after completing one layer, the scanning direction was rotated by 67° for the subsequent layer.

After fabrication, the specimens were removed from the build substrate using electrical discharge machining (EDM, ZE530, Jiangsu Zhengman CNC Equipment Co., Ltd., Taizhou, China) wire cutting to avoid introducing excessive mechanical damage. The samples were subsequently cleaned in an ultrasonic bath with anhydrous ethanol to remove loosely attached or trapped powder particles from both external surfaces and internal pores. Following cleaning, the volume, mass, and apparent density of each specimen were measured to verify consistency with the designed relative densities. Detailed structural and morphological characterization was then conducted using SEM and optical microscopy (OM, TO-HK830), providing a basis for correlating manufacturing quality with the mechanical response and deformation mechanisms analyzed in subsequent sections.

### 2.3. Static Compression Test

Quasi-static uniaxial compression tests were conducted to evaluate the mechanical response of the NiTi porous scaffolds. All tests were performed at room temperature using an MTS microcomputer-controlled electronic universal testing machine (CMT5105, MTS Systems (China) Co., Ltd., Shenzhen, China; maximum load capacity of 100 kN), in accordance with the ISO 13314:2011 standard for compression testing of porous and cellular metals. This standard was selected to ensure consistency and comparability of the obtained mechanical parameters with those reported in the literature for additively manufactured lattice structures.

A constant displacement-controlled loading mode was adopted, with a crosshead speed of 1 mm/min and a data acquisition frequency of 10 Hz. This relatively low loading rate was chosen to minimize dynamic and inertial effects, thereby ensuring that the measured response reflects the quasi-static deformation behavior of the porous scaffolds. Each compression test was continued until either macroscopic structural collapse or complete densification of the specimen occurred, at which point the test was terminated. Throughout the entire loading process, a digital camera was used to continuously record the deformation and failure evolution of the specimens, providing visual evidence for identifying dominant deformation modes and collapse mechanisms discussed later.

To ensure repeatability and statistical reliability, three specimens were tested for each scaffold design, and the reported mechanical properties represent the average values of these measurements. Engineering strain (*ε*) was calculated by dividing the measured axial displacement by the initial height of the specimen, while engineering stress (*σ*) was determined by dividing the applied load by the minimum cross-sectional area perpendicular to the loading direction. This definition of stress is particularly relevant for porous structures, as it enables a more meaningful comparison of load-bearing capacity among scaffolds with different strut sizes and relative densities.

The elastic modulus of the porous scaffolds was extracted from the initial linear region of the stress–strain curve using linear regression, where the slope of the fitted line was taken as Young’s modulus. To characterize compressive strength, two criteria were considered depending on the deformation behavior: (i) the first peak stress observed on the stress–strain curve, corresponding to the onset of structural instability or initial collapse; or (ii) in cases where a pronounced plateau region was present, the average stress within a strain range of 20% to 30%, representing the stable load-bearing capacity during progressive cell collapse. These definitions allow the compressive performance of the scaffolds to be quantitatively compared while capturing both peak-bearing and energy-absorbing characteristics, which are directly related to the deformation and failure mechanisms analyzed in Section 3.

### 2.4. Modeling of Quasi-Static Compression

For porous scaffolds with complex lattice geometries, conventional static implicit analysis may suffer from numerical instability, excessive computational time, and convergence difficulties, particularly when progressive collapse, contact evolution, and localized plasticity occur. In contrast, an explicit dynamic formulation generally provides better robustness for problems involving severe nonlinearity and complex contact, and it can be employed to approximate quasi-static behavior by sufficiently reducing the loading rate and extending the loading duration. In such a quasi-static explicit framework, inertial effects are minimized so that the computed response is dominated by material deformation and contact interactions rather than dynamic vibration.

In the present work, finite element simulations were performed using ABAQUS (ABAQUS 2021, Dassault Systèmes, Vélizy-Villacoublay, France) to investigate the compressive behavior and underlying deformation mechanisms of the NiTi porous scaffolds. The lattice geometries designed in SolidWorks were imported into ABAQUS for preprocessing and analysis. To maintain the model in a quasi-static state while keeping computational cost reasonable, a modal analysis was first conducted, and the cycle time corresponding to the lowest natural frequency was used as a reference to determine an appropriate time scale for the explicit analysis. This strategy enables stable simulations while suppressing spurious dynamic oscillations, which is essential for meaningful comparison with the quasi-static compression tests.

Two levels of numerical models were considered to balance physical fidelity and computational efficiency. First, for parametric assessment of the influence of strut size, single unit-cell models corresponding to each design were simulated to reveal stress localization and deformation features at the cell level. Second, to capture the overall collapse pattern observed experimentally, a full porous scaffold model was established (with particular focus on the specimen with a strut size of 280 μm), enabling direct comparison of macroscopic deformation modes between simulation and experiments.

**Figure 4:**
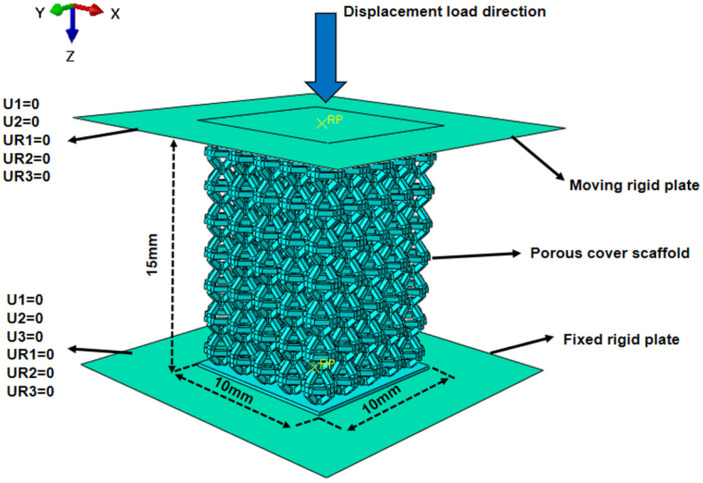
Schematic representation of boundary conditions and loading configuration for the quasi-static compression simulation of the porous scaffold.

The quasi-static compression setup replicated the experimental boundary conditions and consisted of the porous specimen positioned between two rigid plates. The bottom plate was fully fixed, whereas the top plate was constrained to translate only in the vertical (Z) direction. Surface-to-surface contact interactions were defined between the plates and the specimen. Hard contact was imposed in the normal direction, while a tangential friction coefficient of 0.1 was applied to account for interfacial friction effects during compression [29]. The friction coefficient (μ = 0.1) was selected within the commonly adopted range for metal-to-metal contact in quasi-static compression simulations. To evaluate the sensitivity of the predicted deformation mode to this assumption, additional trial simulations were conducted using μ values in a representative range (0.05–0.20). The results indicate that friction mainly affects the degree of deformation asymmetry and the tendency for shear localization near the platen–specimen interface, while the overall collapse-mode classification (progressive layer-by-layer collapse for thinner struts versus shear-band-dominated collapse for thicker struts) remains unchanged within this range. Therefore, μ = 0.1 was retained as a reasonable and physically representative value for the present study. Displacement-controlled loading was adopted, and the top plate was prescribed a vertical displacement corresponding to 60% nominal compressive strain, which was sufficient to trigger progressive collapse and approach the densification regime. The applied boundary conditions are schematically shown in Figure 4.

**Figure 5:**
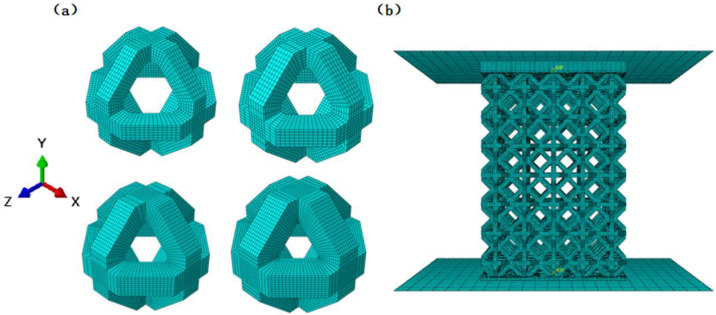
Finite element mesh of the quasi-static compression models: (**a**) single unit cell; (**b**) full-scale porous scaffold with cover plates.

Given the geometric complexity of the lattice architecture, the models were partitioned prior to meshing to improve mesh quality and element regularity. The porous structures were discretized using eight-node linear hexahedral elements with reduced integration (C3D8R), as illustrated in Figure 5. This element type provides a good compromise between computational efficiency and accuracy for large-deformation problems, while reduced integration helps alleviate excessive stiffness in bending-dominated strut responses.

The base material was defined as NiTi with a theoretical density of 6.45 g/cm^3^, Young’s modulus of 19.1 GPa, and Poisson’s ratio of 0.32. To represent the nonlinear plastic behavior of NiTi under compressive loading, the Johnson-Cook constitutive model was adopted in the simulations [30]. This constitutive description enables the model to capture strain-hardening behavior and supports subsequent interpretation of stress redistribution and localization, particularly at nodal junctions where failure is likely to initiate, as discussed in later sections.

It should be noted that the Johnson-Cook model employed in this study does not explicitly account for martensite-austenite phase transformation kinetics inherent to NiTi. However, the present work focuses on monotonic quasi-static compression up to large strains, where the global deformation and collapse sequence are primarily governed by geometric instability, structural load paths, and contact interactions rather than reversible transformation-induced softening. Under these loading conditions, plastic deformation dominates the macroscopic response of the lattice, particularly in the plateau and densification regimes. Therefore, the Johnson-Cook formulation is adopted here as an effective constitutive representation to capture strain hardening and large-deformation behavior in a numerically stable manner. Within this framework, the predicted stress localization and collapse modes are interpreted mainly as geometry-controlled responses of the lattice architecture, rather than as consequences of phase transformation kinetics.

## 3. Results and Discussion

### 3.1. Characterization of Structure and Morphology

**Figure 6:**
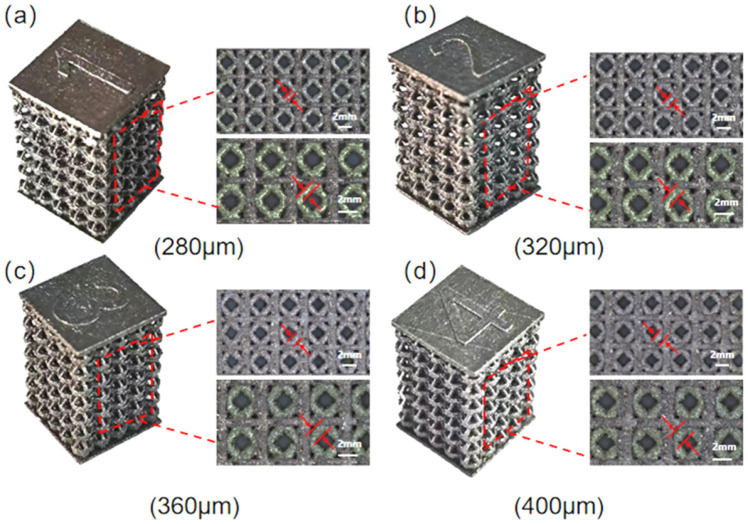
Macroscopic photographs and OM images of PBF-LB/M-fabricated porous scaffolds with different strut sizes: (**a**) 280 μm, (**b**) 320 μm, (**c**) 360 μm, and (**d**) 400 μm.

A systematic characterization of the structural integrity and forming quality of the fabricated specimens is a prerequisite for reliable mechanical testing and subsequent interpretation of deformation behavior. As shown in Figure 6, the porous cover scaffolds fabricated using the optimized PBF-LB/M parameters exhibit excellent dimensional fidelity with respect to their corresponding CAD models. Both direct visual inspection and OM confirm that the overall geometry, external dimensions, and unit-cell arrangement of the printed samples closely match the designed configurations. The scaffold surfaces display a uniform metallic luster and show no macroscopic defects such as visible cracks, severe distortion, or missing strut elements. Furthermore, the variations in strut size among the four scaffold groups are clearly distinguishable, indicating a high level of process stability and repeatability during fabrication.

**Figure 7:**
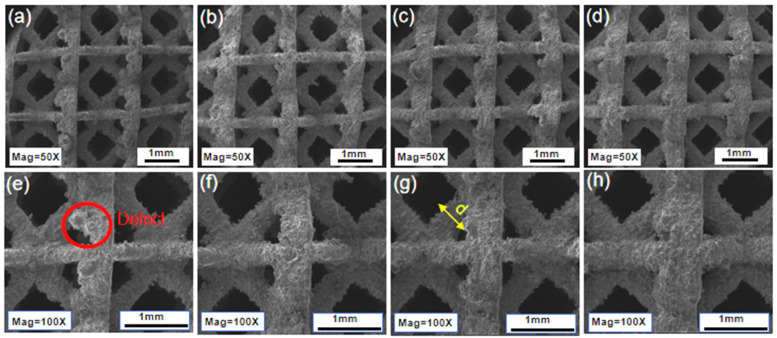
SEM images showing surface morphology of porous scaffolds with different strut sizes at magnifications of 50× and 100×: (**a**,**e**) 280 μm, (**b**,**f**) 320 μm, (**c**,**g**) 360 μm, and (**d**,**h**) 400 μm.

To further evaluate surface quality and local geometric features, detailed surface morphology analyses were performed using SEM. Representative SEM images at magnifications of 50× and 100× are presented in Figure 7. The majority of the struts exhibit smooth and continuous morphologies without evidence of warping, microcracking, or gross deformation, demonstrating a generally high forming quality across all strut sizes. Only a limited number of localized defects are observed, as highlighted by the red circles in Figure 7e. The actual strut diameters are indicated by yellow arrows in the images, confirming that the designed trend in strut size is well preserved after fabrication.

Nevertheless, a noticeable amount of partially melted or unmelted powder particles can be observed adhering to the strut surfaces. This phenomenon is commonly associated with the large temperature gradient between the molten pool and the surrounding loose powder during the PBF-LB/M process. Under such conditions, metal particles may not fully melt and can subsequently adhere to the solidified strut surfaces, leading to a roughened surface morphology [31]. The extent of powder adhesion is not uniform across the structure and appears to be strongly influenced by the inclination angle of individual struts.

In particular, transverse struts exhibit distinct surface features on their upper and lower faces. Unmelted particles and spheroidized features are predominantly found on the upper surfaces, whereas slag accumulation and irregular melt traces are more pronounced on the lower surfaces. During laser scanning, the high-energy beam interacting with the powder bed beneath the transverse struts generates a relatively large molten pool. Due to poor wettability between the molten metal and the surrounding solid powder, spheroidization is promoted, which can locally degrade surface quality and dimensional accuracy. As a result, the effective diameter of transverse struts may slightly exceed their nominal design values. In contrast, the absence of solid support beneath the lower surfaces of overhanging struts leads to direct contact with loose powder, facilitating partial powder detachment during solidification. This mechanism explains the slightly lower measured mass of the printed samples compared with their designed values and the presence of minor internal surface defects [32].

Despite these micro-scale imperfections, the number of defective struts is limited, and their influence on the global mechanical response of the scaffolds is considered negligible. Importantly, no large-scale structural discontinuities or systematic defects are observed that could dominate the compressive behavior. Overall, the PBF-LB/M-fabricated NiTi porous scaffolds accurately reproduce the intended geometric design and enable the precise and orderly construction of complex lattice architectures. Compared with conventional manufacturing techniques, additive manufacturing demonstrates a clear advantage in producing architected porous structures with high geometric complexity and controlled features across both macro- and micro-scales [33], thereby providing a reliable structural basis for the subsequent compression tests and numerical simulations.

### 3.2. Quasi-Static Compression Response

#### 3.2.1. Stress–Strain Curves

**Figure 8:**
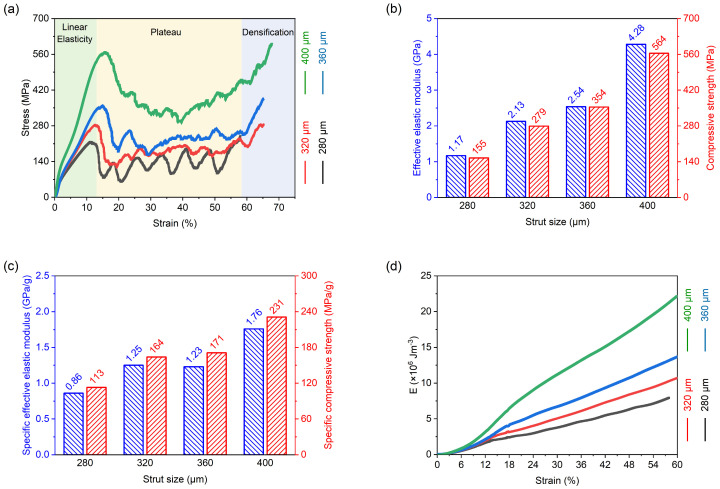
Quasi-static compressive response of porous scaffolds with different strut sizes: (**a**) stress–strain curves; (**b**) effective elastic modulus and compressive strength; (**c**) specific effective elastic modulus and specific compressive strength; (**d**) cumulative energy absorption–strain curves.

Figure 8a presents the quasi-static compressive stress–strain responses of the PBF-LB/M-fabricated NiTi octahedral porous scaffolds with different strut sizes. Despite the variation in geometric parameters, all scaffolds exhibit qualitatively similar stress–strain characteristics, which can be divided into three distinct deformation stages as the applied strain increases [34,35]. This consistency reflects the common octahedral topology employed in all specimens, while the quantitative differences arise primarily from variations in strut dimension and relative density.

The initial stage corresponds to linear-elastic deformation, in which the compressive stress increases approximately linearly with strain. This behavior is dominated by elastic bending and stretching of the load-bearing struts within the octahedral lattice. In this regime, the structural response is stable, and no visible damage or permanent deformation is observed. The slope of the stress–strain curve in this region reflects the effective elastic stiffness of the porous scaffold, which is strongly influenced by strut diameter and the associated load-bearing cross-sectional area.

Following the elastic regime, the scaffolds enter the plastic plateau stage, where the stress reaches a peak value and subsequently exhibits a fluctuating or oscillatory response with increasing strain. This stage is associated with the onset of cell yielding and localized strut failure, leading to irreversible plastic deformation and significant energy absorption. The amplitude and regularity of the stress oscillations are highly dependent on the scaffold geometry. In particular, thinner struts tend to promote more pronounced stress fluctuations, whereas thicker struts result in a smoother plateau response. These oscillations are indicative of sequential cell collapse events rather than uniform deformation, a behavior that is further examined through deformation observations and finite element analysis in subsequent sections.

At higher strain levels, the scaffolds transition into the densification stage, where previously deformed and fractured struts come into contact and compact against each other. This contact-driven constraint leads to a rapid increase in compressive stress, and the stress–strain response gradually approaches that of bulk material compression. The onset strain of densification is influenced by the initial relative density and strut size, with denser structures entering this regime at lower strains.

Although the overall shapes of the stress–strain curves are similar across all specimens, clear quantitative differences are observed in the peak stress values attained during the elastic-to-plastic transition. Specifically, the maximum stress increases monotonically with increasing strut diameter. A particularly notable behavior is observed for the scaffold with a strut size of 280 μm, which exhibits pronounced and periodic stress oscillations characterized by alternating peaks and troughs throughout the plateau region. Each oscillation cycle corresponds to the collapse of a single layer of unit cells, indicating a layer-by-layer collapse mechanism. This behavior contrasts with the smoother responses observed in scaffolds with larger strut sizes and foreshadows the transition in macroscopic failure modes discussed later.

To quantitatively compare the mechanical performance of the different scaffold designs, the elastic modulus was extracted from the slope of the stress–strain curve in the linear-elastic region, while the compressive strength was defined as the first peak stress on the curve. It should be noted that the modulus reported here is an effective (apparent) elastic modulus of the porous scaffold, extracted from the initial linear region of the scaffold stress–strain curve. This parameter reflects the elastic structural stiffness of the lattice architecture rather than the intrinsic Young’s modulus of bulk NiTi. The resulting values for the four strut sizes are summarized in Figure 8b. For strut diameters of 280 μm, 320 μm, 360 μm, and 400 μm, the elastic moduli are 1.17 GPa, 2.13 GPa, 2.54 GPa, and 4.28 GPa, respectively, while the corresponding compressive strengths are 155 MPa, 279 MPa, 354 MPa, and 564 MPa. A pronounced increase in both elastic modulus and compressive strength is observed when the strut size increases from 360 μm to 400 μm, with increments of approximately 68% and 59%, respectively. This nonlinear enhancement suggests a threshold effect associated with increased load-bearing cross-sectional area and reduced susceptibility to localized instability. These trends are consistent with previous reports on octahedral lattice structures, which show that, for a fixed unit-cell size, both stiffness and strength scale positively with strut diameter [36].

However, the primary advantage of lattice structures lies in achieving a balance between low weight and mechanical strength rather than maximizing absolute stiffness or strength. Therefore, to further evaluate the weight-efficiency of the NiTi porous scaffolds, specific effective elastic modulus and specific compressive strength were introduced as normalized performance indicators. The specific effective elastic modulus was calculated by dividing the effective elastic modulus by the mass of the porous region, while the specific compressive strength was defined as the compressive strength divided by the mass of the porous region. The corresponding results are presented in Figure 8c. As shown in Figure 8c, both the specific effective elastic modulus and specific compressive strength exhibit trends similar to those observed for the absolute effective modulus and compressive strength in Figure 8b. Specifically, these normalized parameters generally increase with increasing strut size.

This trend can be understood from the perspective of relative density and load transfer mechanisms in stretch-dominated lattice structures. According to the Gibson–Ashby model for cellular solids, the elastic modulus and strength of stretch-dominated lattices scale approximately linearly with relative density, whereas bending-dominated structures follow higher-order power-law relations. The ortho-octahedral topology employed in this study belongs to a stretch-dominated architecture, where load is primarily transmitted along the axial direction of the struts. As the strut size increases, the relative density increases, leading to enhanced load-carrying capacity and improved stiffness efficiency. Although the mass of the porous region also increases with strut size, the improvement in structural load-bearing efficiency outweighs the mass increment, resulting in a gradual increase in specific mechanical properties. These results indicate that within the investigated range, increasing strut size enhances not only absolute mechanical performance but also structural efficiency under weight constraints, which is critical for lightweight biomedical and load-bearing applications.

In addition to strength and stiffness, energy absorption was evaluated by integrating the compressive response up to a prescribed strain level (i.e., the area under the stress–strain curve). The cumulative energy absorption–strain curves of NiTi porous scaffolds with different strut sizes are shown in Figure 8d. The cumulative energy absorption increases continuously with strain for all scaffolds. A clear dependence on strut size is observed: scaffolds with larger strut sizes exhibit higher cumulative energy absorption throughout the investigated strain range. The differences in energy absorption behavior are closely related to the strain-dependent deformation mechanisms identified in the macroscopic observations. For the small-strut scaffold, deformation proceeds predominantly via progressive layer-by-layer collapse, which is manifested by the oscillatory plateau on the stress–strain curves. This sequential crushing mode enables sustained energy dissipation through repeated local yielding and progressive densification of individual layers. In contrast, scaffolds with larger strut sizes tend to develop a shear-dominated failure band along an approximately 45° plane at later stages of compression. Such localization indicates that deformation becomes concentrated in a preferred shear path, leading to the formation of a macroscopic collapse band rather than a strictly layer-wise crushing process.

From the perspective of the Gibson–Ashby framework, the energy absorption capability of cellular solids is governed by both relative density and deformation mode. Increasing strut size increases the relative density and the load-bearing cross-sectional area of the struts, thereby elevating the characteristic stress level that the lattice can sustain during compression. Consequently, even though the deformation of large-strut scaffolds becomes more localized through shear-band formation, the higher stress carried during the elastic and plateau stages results in a larger accumulated area under the stress–strain curve, and thus higher cumulative energy absorption. Meanwhile, the small-strut scaffold dissipates energy in a more distributed and progressive manner through layer-by-layer collapse, which contributes to stable energy absorption with pronounced stress oscillations. Therefore, within the investigated design space, strut size influences not only the magnitude of energy absorption (through density-dependent stress scaling), but also the dominant dissipation pathway (distributed progressive crushing versus localized shear-band collapse). These findings highlight the importance of simultaneously considering relative density and deformation mode when optimizing NiTi porous scaffolds for energy-dissipative and load-bearing applications.

#### 3.2.2. Deformation Mechanisms

**Figure 9:**
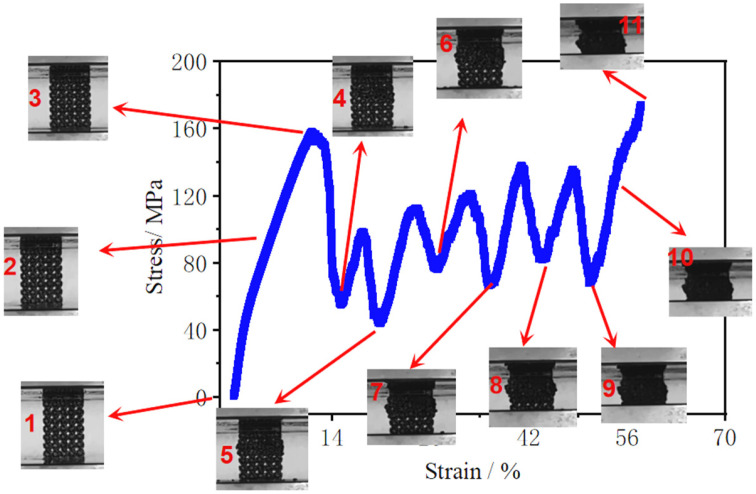
Progressive layer-by-layer deformation and collapse behavior of the porous scaffold under quasi-static (The numbered images indicate the deformation states at increasing strain levels).

**Figure 10:**
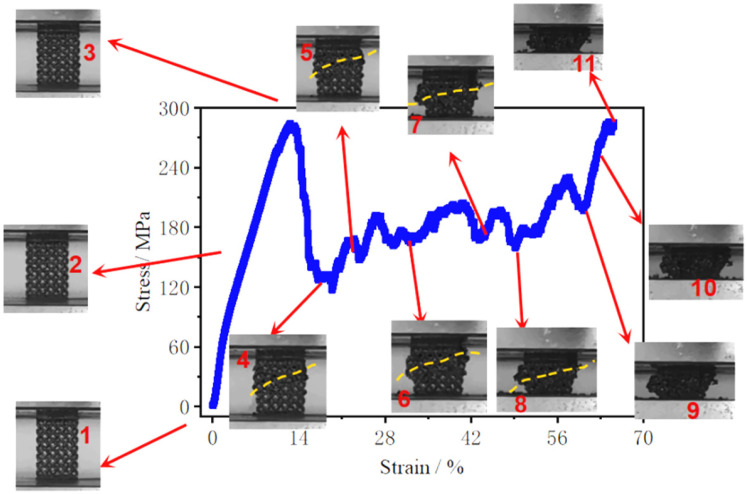
Macroscopic deformation pattern characterized by shear-dominated failure along an approximately 45° fracture plane (The numbered images indicate the deformation states at increasing strain levels).

The macroscopic deformation and failure evolution of the porous scaffolds under compressive loading are summarized in Figure 9 and Figure 10. Two dominant deformation modes can be identified: progressive layer-by-layer crushing and 45° shear-band dominated fracture. Although both modes initiate with a similar elastic response, they diverge markedly after yielding, consistent with the distinct stress–strain features reported in Section 3.2.1.

For the layer-by-layer crushing mode (Figure 9), the specimens exhibit stable deformation at the early stages of loading (inserts 1–3), where no obvious fracture is observed and only slight outward bulging develops in the mid-height region. This bulging is a typical response of cellular lattices under uniaxial compression and reflects localized bending and rotation of struts as the structure transitions toward plastic collapse. With continued loading, fracture initiates locally and the first layer of unit cells begins to collapse (inserts 4–8). The collapsed layer subsequently compacts and provides a relatively rigid support for the next layer, leading to a sequential, layer-by-layer collapse process. This progressive crushing continues until most layers have densified and the specimen approaches global compaction, consistent with the periodic stress oscillations observed for the thinner-strut scaffold in Figure 8a. Importantly, such a deformation pathway suggests that collapse is controlled by localized instability within discrete cell layers, rather than by a single catastrophic shear event.

In contrast, Figure 10 shows the deformation mode characterized by a distinct fracture zone inclined at approximately 45° to the loading direction (highlighted by the yellow dotted line). Similar to the layer-by-layer mode, the early deformation stages (inserts 1–3) are dominated by minor bulging and overall compression without obvious cracking. As strain increases, however, damage localizes along an oblique band, and strut failure propagates preferentially across adjacent layers. This results in the formation of a macroscopic shear plane and a more rapid loss of structural integrity compared with progressive crushing. The development of a 45° shear band indicates that the scaffold deformation becomes governed by combined compressive and shear stresses, which may be promoted by geometric constraints, frictional boundary conditions at the platen-specimen interfaces, and the redistribution of load paths as local collapse initiates. The transition from progressive crushing to shear-dominated collapse is therefore closely linked to the interplay between lattice geometry (strut size and connectivity) and the evolving internal stress field—an aspect further clarified by the stress localization results obtained from finite element simulations. From a strut-level stability perspective, this transition can also be rationalized by a buckling-to-yield competition. When the struts are relatively thin, the higher effective slenderness makes local bending and instability more favorable, which promotes distributed collapse and progressive layer-by-layer crushing. As the strut diameter increases, the effective slenderness decreases and the strut-level yield/bending resistance increases, reducing the propensity for distributed buckling and encouraging deformation localization under combined compressive-shear stresses. As a result, the failure mode evolves toward a shear-band-dominated collapse characterized by an approximately 45° fracture plane. We note that establishing a quantitative critical slenderness threshold would require dedicated instability analysis; nevertheless, the present observations consistently support the role of geometry-dependent stability in governing the collapse-mode transition, and this valuable aspect will be investigated more systematically in our future work.

During compression, small fragments were occasionally observed to detach from the specimens. This phenomenon can be attributed to the brittle separation of locally weakened struts and the progressive dislodgement of partially bonded or defect-containing regions under sustained loading. Such debris formation is consistent with the presence of microstructural imperfections and unmelted powder attachments identified in the surface morphology analysis.

**Figure 11:**
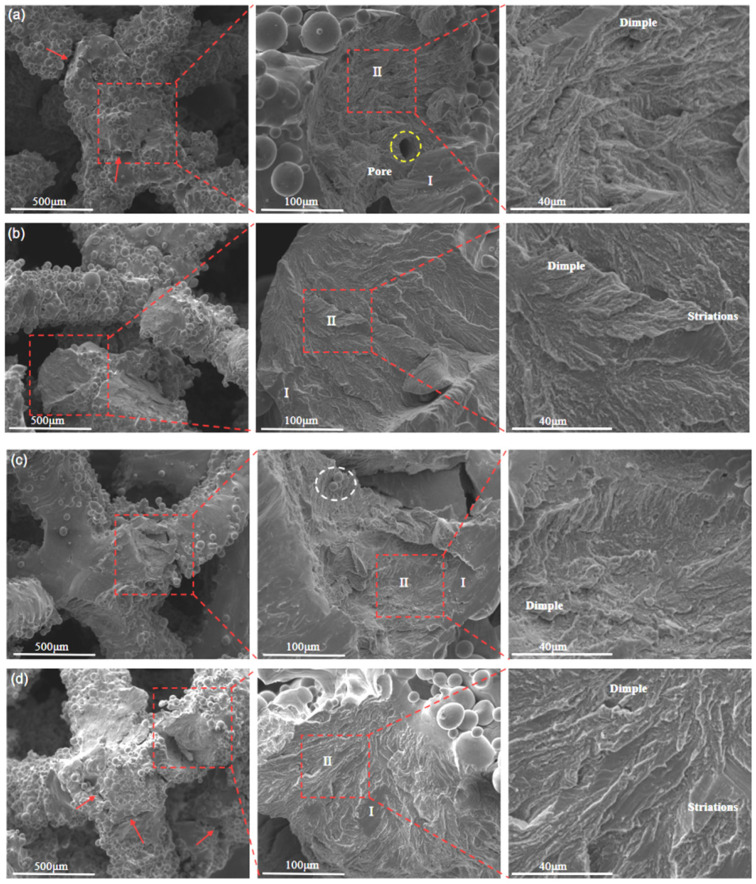
SEM images of fracture surfaces after compression for porous scaffolds with different strut sizes: (**a**) 280 μm, (**b**) 320 μm, (**c**) 360 μm, and (**d**) 400 μm (The red arrows indicate crack locations).

To elucidate the underlying failure mechanisms, fracture surfaces were examined by SEM, with representative morphologies shown in Figure 11. The fracture surfaces typically exhibit a cup-like macroscopic profile accompanied by pronounced surface irregularities. Voids are visible in Figure 11a (yellow dashed circles), while unmelted or partially melted powder particles are evident in Figure 11c (white dashed circles). In laser-melted materials, these unmelted-particle regions and associated surface defects act as preferential sites for stress concentration, thereby promoting crack initiation and reducing the extent of stable plastic deformation prior to failure. While internal pores may locally reduce the load-bearing capacity of struts, the observations suggest that they are not the primary triggers for crack nucleation in the present structures.

A key feature revealed by Figure 11d is that multiple crack initiation sites are concentrated at the nodal junctions of the lattice (red arrows). Moreover, the majority of observed crack initiation and subsequent fracture events are localized at these junction regions, indicating that failure preferentially initiates at the scaffold nodes. This is consistent with the geometric nature of lattice structures, where nodal intersections experience elevated stress triaxiality and bending moments, and is further reinforced by local geometric irregularities caused by adhered unmelted powder particles that can create sharp corners and notch-like features. These experimental observations directly support the later numerical findings that stress concentration is most pronounced at nodal junctions. Overall, the preferential crack initiation at nodal junctions is attributed to a combined effect of geometry-driven stress amplification (load-path convergence and locally elevated multiaxial stress states) and process-induced defect amplification (surface roughness, adhered/unmelted particles, and notch-like irregularities formed during PBF-LB/M). Although the present observations and simulations consistently indicate node-dominated stress localization, a quantitative decoupling of these two contributions would require controlled surface modification (e.g., polishing/blasting) or a systematic tailoring of defect populations while maintaining identical lattice geometry.

In the crack propagation regions, irregular cross-sections and secondary microcracks are frequently observed, reflecting the mixed crack-growth behavior of additively manufactured NiTi [37]. Two characteristic zones can be distinguished on the fracture surface: a relatively smooth zone (Region I) and a rougher zone (Region II) containing fine and coarse dimples. In addition, randomly distributed micropores and strip-like features are present. At higher magnification, river-like patterns and tear ridges (e.g., Figure 11b,d) indicate that a certain degree of plastic deformation occurred prior to final separation, which is typical of ductile fracture. Meanwhile, locally flat facets are also observed, suggesting a concurrent brittle fracture contribution. Therefore, the overall fracture behavior can be categorized as a mixed ductile-brittle fracture mode, consistent with fracture characteristics previously reported for additively manufactured metallic scaffolds [38].

### 3.3. Finite Element Analysis

#### 3.3.1. Quasi-Static Single-Cell Finite Element Analysis

Quasi-static uniaxial compression simulations were performed for four single-unit-cell models with different strut sizes, using the same boundary/contact definitions and loading scheme as those adopted for the scaffold-level analyses. In all cases, the models were compressed to a nominal strain of 60%, which is sufficient to capture the elastic response, post-yield collapse behavior, and the onset of densification at the cell level.

Because the simulations were conducted using ABAQUS/Explicit, it is necessary to confirm that the computed response corresponds to a quasi-static process rather than a dynamically dominated event. This verification was performed by examining the energy evolution and the overall energy balance of the finite element system, which can be expressed as:

(1) $$E_{TOT}=E_{I}+E_{KE}+E_{FD}+E_{V}-E_{W}=Cons\tan t$$

where *E_I_* represents the internal energy, including both elastic and plastic strain energy, *E_V_* denotes the energy absorbed due to viscous dissipation, *E_KE_* is the kinetic energy, *E_FD_* is the energy absorbed through frictional dissipation, *E_W_* is the work done by the external force, and *E_TOT_* is the total energy in the system.

**Figure 12:**
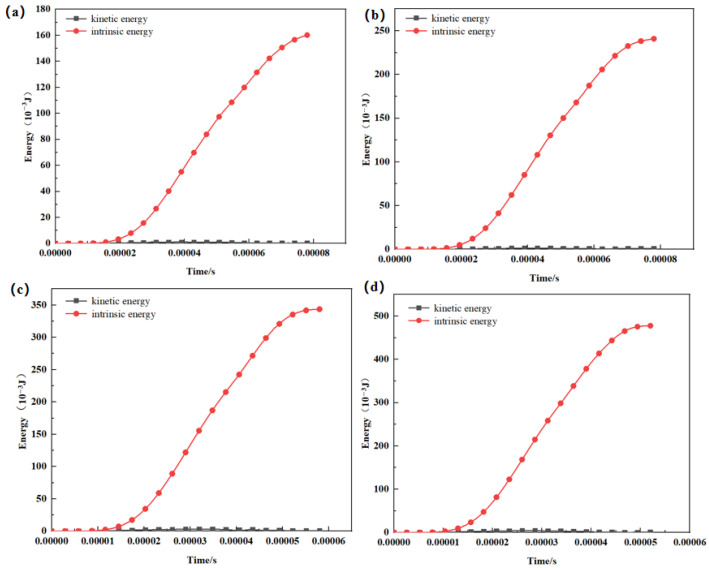
Energy evolution during quasi-static compression simulations of single-unit-cell models with different strut sizes: (**a**) 280 μm, (**b**) 320 μm, (**c**) 360 μm, and (**d**) 400 μm.

In an explicit quasi-static simulation, the external work is expected to closely track the total system energy, while inertial effects remain small because material velocities are negligible. Accordingly, the kinetic energy should be minimal throughout loading. A commonly accepted criterion is that, under quasi-static conditions, the kinetic energy should not exceed approximately 5–10% of the internal energy during deformation [39]. As shown by the energy curves in Figure 12, this condition is satisfied for all four single-cell models, confirming that the simulations reliably represent quasi-static compression.

ABAQUS/Explicit does not directly output a conventional engineering stress–strain curve for the unit-cell model. Therefore, the nodal reaction force at the constrained support and the corresponding displacement were first extracted to obtain the load–displacement response. The load–displacement data were then converted into engineering stress and strain using:

(2) $$\sigma =\frac{F}{S}$$

(3) $$\varepsilon =\frac{\Delta h}{h}$$

where *σ* denotes stress, *F* represents the nodal support reaction force, *S* indicates the minimum cross-sectional area of a single cell, *ɛ* signifies strain, ∆*h* refers to the compression displacement, and *h* is the initial height of the single cell model.

**Figure 13:**
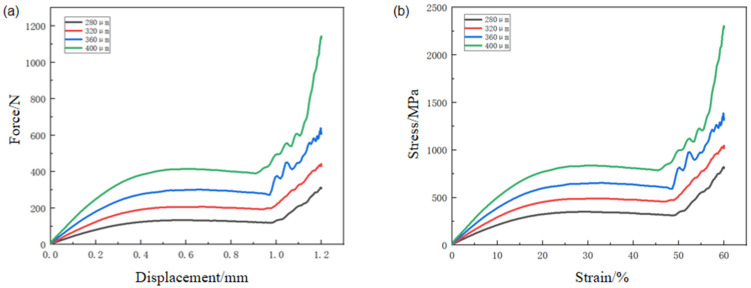
Simulation-derived mechanical response of single-unit-cell models with different strut sizes: (**a**) force–displacement curves; (**b**) corresponding stress–strain curves.

The resulting stress–strain curves are presented in Figure 13 and exhibit consistent trends across all strut sizes. With increasing strut diameter, the stress level increases over the entire deformation range, indicating enhanced stiffness and load-carrying capability at the cell scale—consistent with the experimental observations at the scaffold level.

**Figure 14:**
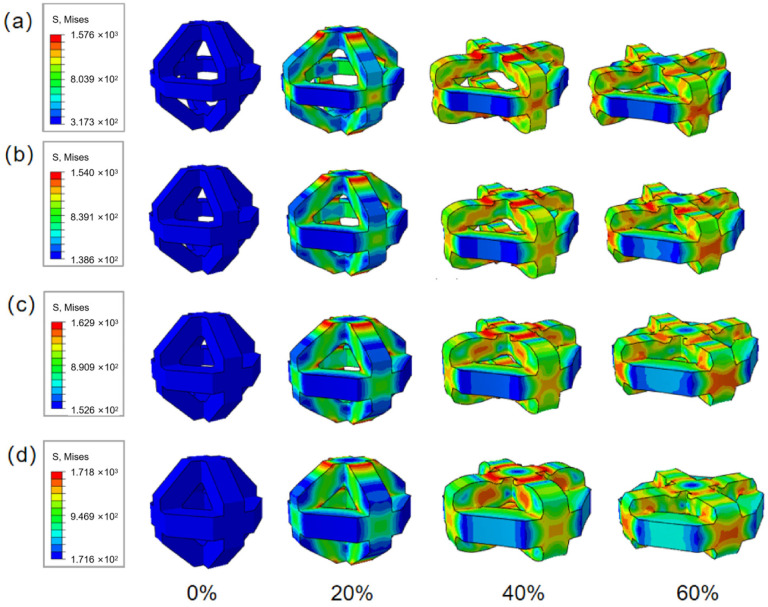
Stress distribution contours and deformation states of single-unit-cell models under quasi-static compression: (**a**) 280 μm, (**b**) 320 μm, (**c**) 360 μm, and (**d**) 400 μm.

Figure 14 shows representative stress contours and deformation states of the four unit-cell models during compression. A pronounced feature common to all cases is the development of stress concentration at the nodal junctions, where multiple struts intersect. This localization is consistent with the experimentally observed crack initiation sites at junction regions and supports the interpretation that node-dominated stress amplification governs the onset of damage. In addition, the compressive load is primarily transferred along struts aligned with the loading direction, whereas struts oriented more horizontally experience comparatively lower stress levels, reflecting the topology-dependent load path within the octahedral unit cell [40,41].

From a geometric perspective, the ability of a lattice cell to sustain compressive loading is strongly influenced by the effective cross-sectional area perpendicular to the loading direction. In general, a larger load-bearing area enables higher sustainable compressive/tensile stress before localized instability or failure occurs [42]. Based on this principle, the minimum cross-sectional area normal to the loading direction under maximum stress was evaluated for each unit-cell design, yielding 0.3808 mm^2^, 0.4224 mm^2^, 0.4608 mm^2^, and 0.4960 mm^2^ for strut sizes of 280 μm, 320 μm, 360 μm, and 400 μm, respectively. The 400 μm unit cell therefore provides the largest effective load-bearing area, which facilitates more efficient stress transfer in the loading direction and offers a mechanistic explanation for its markedly higher compressive strength observed experimentally.

#### 3.3.2. Analyses of Simulation-Controlled Tests

To further validate the reliability of the numerical framework and its ability to capture the experimentally observed deformation mechanisms, a quasi-static uniaxial compression simulation up to 60% strain was performed on a full-scale porous scaffold with a strut size of 280 μm. This scaffold configuration was selected because it exhibits the most pronounced layer-by-layer collapse behavior and stress oscillations in the experimental stress–strain response, making it particularly suitable for assessing the predictive capability of the finite element model.

**Figure 15:**
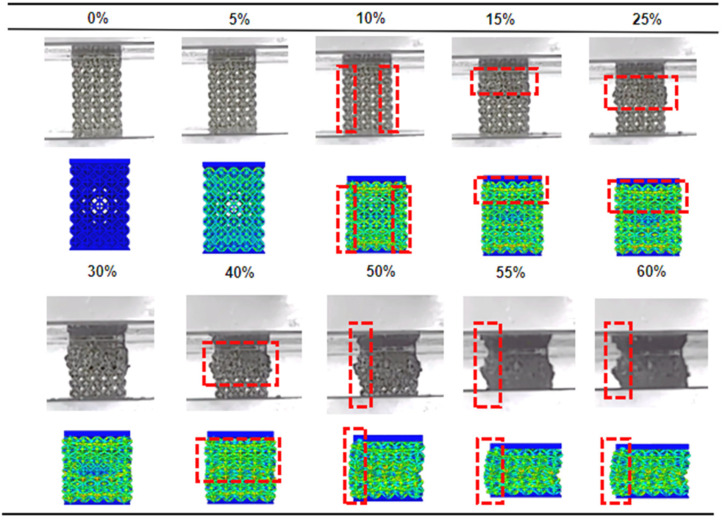
Comparison between experimental observations and finite element simulation results for the porous scaffold with a strut size of 280 μm under quasi-static compression (The red dashed boxes highlight the regions exhibiting significant deformation).

A direct comparison between the numerical simulation and the corresponding experimental compression test is presented in Figure 15. At the initial stage of loading, both the simulation and experimental results show stable deformation without visible fracture, accompanied by a slight outward expansion in the central region of the scaffold. This behavior reflects the elastic bending and rotation of struts prior to the onset of localized failure. As compression proceeds, fracture initiates in the uppermost cell layer, followed by progressive crushing and compaction of the first and second layers. Once the upper collapsed layer comes into contact with the underlying intact layer, further loading triggers subsequent layer collapse, resulting in a sequential, layer-by-layer densification process. This simulated collapse sequence closely reproduces the experimentally observed rod fracturing and block-like fragmentation, although the apparent splattering of fragments in the experimental images is partly influenced by the limited camera viewing angle during testing.

As compression continues, the scaffold undergoes progressive densification until most of the cellular voids are eliminated and the structure approaches a compacted state, consistent with the final stage of the experimental stress–strain curves [43]. Importantly, a similar asymmetric outward extrusion is observed in both simulation and experiment, where the left side of the scaffold exhibits localized outward extrusion and expansion. This consistency supports the use of the adopted friction coefficient in reproducing the experimentally observed deformation tendency, although the dominant collapse mechanisms remain primarily governed by lattice architecture and relative density. As a result, the deformation becomes non-uniform along the height of the scaffold, with greater lateral expansion occurring away from the constrained region. Importantly, this asymmetry is not an artifact of numerical modeling but rather a physically meaningful consequence of boundary friction, further reinforcing the realism of the simulation setup.

Overall, the close agreement between simulated and experimental deformation patterns, including the onset of bulging, the sequence of layer collapse, and the influence of friction-induced constraints, demonstrates that the finite element model accurately captures the essential damage and collapse characteristics of the NiTi porous scaffold under quasi-static compression. This consistency validates the predictive reliability of the numerical approach and supports its use as an effective tool for analyzing stress localization, deformation mechanisms, and structure-property relationships in architected porous scaffolds.

## 4. Conclusions

In this work, NiTi ortho-octahedral porous scaffolds with four different strut sizes were successfully fabricated by PBF-LB/M. The compressive behavior, deformation evolution, and failure mechanisms of the scaffolds were systematically investigated through a combination of quasi-static compression experiments and finite element simulations. Based on the experimental observations and validated numerical results, the main conclusions are summarized as follows:

(1) NiTi ortho-octahedral porous scaffolds with four strut sizes were successfully fabricated via selective laser melting, exhibiting good dimensional fidelity to the CAD models and stable build quality across the investigated parameter range. The printed lattices maintained clear strut-size differentiation with no macroscopic defects that would dominate the compressive response, providing a reliable structural basis for mechanical evaluation.
(2) All scaffolds displayed the typical three-stage compressive response of cellular metals (linear-elastic regime, post-yield plateau, and densification). Increasing strut size markedly enhanced both stiffness and strength: the elastic modulus increased from 1.17 GPa to 4.28 GPa and the compressive strength increased from 155 MPa to 564 MPa as strut size increased from 280 μm to 400 μm. In parallel, the macroscopic deformation mode evolved from a progressive layer-by-layer collapse (pronounced stress oscillations for 280 μm) to a shear-band-dominated failure characterized by an ~45° fracture zone for larger struts.
(3) Explicit quasi-static finite element simulations reproduced the experimentally observed collapse sequence and confirmed that stress is preferentially localized at nodal junctions of the unit cells. Load transfer was dominated by struts aligned with the loading direction, while horizontally oriented members carried comparatively lower stresses. The increase in effective load-bearing cross-sectional area with strut size provides a direct geometric mechanism for the observed strength enhancement, with the 400 μm design showing the largest cross-sectional area and the highest compressive capacity.

## Figures and Tables

**Table 1:** Geometric parameters of ortho-octahedral unit cells with different strut sizes.

Serial Number	Strut Size	Cell Size	Cell Volume	Relative Density	Number of Individuals	Size/mm	Cross-Section
	μm	mm	mm^3^	%	x-y-z	x-y-z	mm^2^
1	280	2	1.21	15.13	5-5-7	10-10-14	9.52
2	320	2	1.51	18.88	5-5-7	10-10-14	10.56
3	360	2	1.83	22.88	5-5-7	10-10-14	11.52
4	400	2	2.16	27.00	5-5-7	10-10-14	12.40

## Data Availability

The original contributions presented in this study are included in the article. Further inquiries can be directed to the corresponding author.

## References

[1] Liu H.S., Ge J.G., Zhao G.B., Chen Y., Yue G., He S.L., Zhan W.W., Zhang Y.H., Liu Z.M. (2024). An investigation on microstructural characteristics and comprehensive performances of equiatomic ratio NiTi shape-memory alloy produced by selective laser melting. Adv. Eng. Mater..

[2] Bansiddhi A., Sargeant T.D., Stupp S.I., Dunand D.C. (2008). Porous NiTi for bone implants: A review. Acta Biomater..

[3] Zhao M., Qing H.B., Wang Y.X., Liang J., Zhao M.Y., Geng Y.L., Liang J.Z., Lu B.H. (2021). Superelastic behaviors of additively manufactured porous NiTi shape memory alloys designed with Menger sponge-like fractal structures. Mater. Des..

[4] Li S., Zhang H., Li S., Wang J.Q., Wang Q.W., Cheng Z.L. (2024). Advances in hierarchically porous materials: Fundamentals, preparation and applications. Renew. Sustain. Energy Rev..

[5] Li Y.H., Shang X.Y. (2020). Recent progress in porous TiNb-based alloys for biomedical implant applications. Mater. Sci. Technol..

[6] Chen D.Z., Gong W.T., Xu C., Zhang Y.J., Hao R., Yang Z.B., Chen R.R., Fu H.Z. (2025). Oxidation behavior of Ge-modified Nb-Si alloys at 1250 °C: Self-generated NbGe_2_ layer with enhanced oxidation resistance. Appl. Surf. Sci. Adv..

[7] Xiong Z.W., Li Z.H., Sun Z., Hao S.J., Yang Y., Li M., Song C.H., Qiu P., Cui L.S. (2019). Selective laser melting of NiTi alloy with superior tensile property and shape memory effect. J. Mater. Sci. Technol..

[8] Xu K.K., Gong Y.D., Zhao Q.Z. (2023). Comparison of traditional processing and additive manufacturing technologies in various performance aspects: A review. Arch. Civ. Mech. Eng..

[9] Sabban R., Bahl S., Chatterjee K., Suwas S. (2018). Globularization using heat treatment in additively manufactured Ti-6Al-4V for high strength and toughness. Acta Mater..

[10] Qiu C.L., Adkins N.J.E., Attallah M.M. (2013). Microstructure and tensile properties of selectively laser-melted and HIPed laser-melted Ti-6Al-4V. Mater. Sci. Eng. A.

[11] Mishra A.K., Kumar A. (2019). Numerical and experimental analysis of the effect of volumetric energy absorption in powder layer on thermal-fluidic transport in selective laser melting of Ti-6Al-4V. Opt. Laser Technol..

[12] Zhang Y.M., Huang W.B. (2020). Comparisons of 304 austenitic stainless steel manufactured by laser metal deposition and selective laser melting. J. Manuf. Process..

[13] Chen D.Z., Wang Y.X., Yu J.Y., Xu C., Wang D.X., Dai G.X., Xu Q., Wang Q., Chen R.R. (2025). Er-Induced Microstructural Refinement and Internal Oxidation Enhancing Fracture Toughness in Nb-Si Alloys. J. Alloys Compd..

[14] Li R.D., Niu P.D., Yuan T.C., Cao P., Chen C., Zhou K.C. (2018). Selective laser melting of an equiatomic CoCrFeMnNi high-entropy alloy: Processability, non-equilibrium microstructure and mechanical property. J. Alloys Compd..

[15] Zhang Y., Shi X.Z., Du Z.Q., Yang Y.H., Liu X.C., Li Y.L., Shen J.H. (2023). Microstructural strengthening and plastic degradation of Ti-6Al-4V induced by laser ablation. Met. Mater. Int..

[16] Sallica-Leva E., Jardini A.L., Fogagnolo J.B. (2013). Microstructure and mechanical behavior of porous Ti-6Al-4V parts obtained by selective laser melting. J. Mech. Behav. Biomed. Mater..

[17] Bormann T., Schumacher R., Müller B., Mertmann M., Wild M. (2012). Tailoring selective laser melting process parameters for NiTi implants. J. Mater. Eng. Perform..

[18] Dadbakhsh S., Speirs M., Kruth J.P., Schrooten J., Luyten J., Van Humbeeck J. (2014). Effect of SLM parameters on transformation temperatures of shape memory nickel titanium parts. Adv. Eng. Mater..

[19] Habijan T., Haberland C., Meier H., Frenzel J., Wittsiepe J., Wuwer C., Greulich C., Schildhauer T.A., Köller M. (2013). The biocompatibility of dense and porous nickel–titanium produced by selective laser melting. Mater. Sci. Eng. C.

[20] Liu S., Guo R.Q., Niu H.Z., Wang X.P., Yang J.H., Zhang D.L. (2024). Mechanical properties and energy absorption characteristics of additively manufactured lattice structures of a high-temperature titanium matrix composite. Adv. Eng. Mater..

[21] Gao Y., Liu B., Hao Y.B., Li Z.H., Li Y.D., Guo X.T., Zhang F.Y., Zhang K.F. (2024). Effect of cell size on the mechanical properties of the porous structure of a CuCrZr alloy formed by selective laser melting technology. Adv. Eng. Mater..

[22] Li Z., Mo H.T., Tian J.H., Li J.H., Xia S.Q., Jia X.S., Zhou L.B., Lu Y. (2024). Compressive properties and fracture behaviours of Ti/Al interpenetrating phase composites with additively manufactured TPMS porous structures. Met. Mater. Int..

[23] Fan Z.F., Huang G.H., Lu Y.J., Yan C., Zeng F.Y., Lin J.X. (2022). Full compression response of FG-based scaffolds with varying porosity via an effective numerical scheme. Int. J. Mech. Sci..

[24] Fousová M., Vojtěch D., Kubásek J., Jablonská E., Fojt J. (2017). Promising characteristics of gradient porosity Ti-6Al-4V alloy prepared by SLM process. J. Mech. Behav. Biomed. Mater..

[25] Huang X., Ding S.B., Lang L.H., Gong S.L. (2023). Compressive response of selective laser-melted lattice structures with different strut sizes based on theoretical, numerical and experimental approaches. Rapid Prototyp. J..

[26] Weaire D., Fortes M.A. (2006). Stress and strain in liquid and solid foams. Adv. Phys..

[27] Zhang G.Q., Li J.X., Li J., Zhang C.G., Xiao Z.F. (2018). Simulation analysis and performance study of CoCrMo porous structure manufactured by selective laser melting. J. Mater. Eng. Perform..

[28] Ge J.G., Yuan B., Zhao L., Yan M., Chen W., Zhang L. (2022). Effect of volume energy density on selective laser melting NiTi shape memory alloys: Microstructural evolution, mechanical and functional properties. J. Mater. Res. Technol..

[29] Bai L., Gong C., Chen X.H., Zheng J., Xin L.M., Xiong Y., Wu X.Y., Hu M.J., Li K., Sun Y.X. (2021). Quasi-static compressive responses and fatigue behaviour of Ti-6Al-4V graded lattice structures fabricated by laser powder bed fusion. Mater. Des..

[30] Jalali M., Mohammadi K., Movahhedy M.R., Karimi F., Sadmezhaad S.K., Chemyshikhin S.V., Shishkovsky L.V. (2023). SLM additive manufacturing of NiTi porous implants: A review of constitutive models, finite element simulations, manufacturing, heat treatment, mechanical, and biomedical studies. Met. Mater. Int..

[31] Bai L., Xu Y., Chen X.H., Xin L.M., Zhang J.F., Li K., Sun Y.X. (2021). Improved mechanical properties and energy absorption of Ti-6Al-4V LPBF lattice structures using curving lattice struts. Mater. Des..

[32] Wang P., Yang F., Lu G.X., Bian Y.J., Zhang S.Y., Zheng B.L., Fan H.L. (2022). Anisotropic compression behaviors of bio-inspired modified body-centered cubic lattices validated by additive manufacturing. Compos. Part B Eng..

[33] Yan S.T., Li Y., Tian Z.F., Ming W.J., Wang X.L. (2021). A review of additive manufacturing technology and its application to foundry in China. China Foundry.

[34] Ge J.G., Yan X.C., Lei Y.P., Ahmed M., Reilly P., Zhang C., Lupoi R., Yin S. (2020). A detailed analysis on the microstructure and compressive properties of selective laser melted Ti-6Al-4V lattice structures. Mater. Des..

[35] Yang L., Mertens R., Ferrucci M., Yan C.Z., Shi Y.S., Yang S.F. (2019). Continuous graded gyroid cellular structures fabricated by selective laser melting: Design, manufacturing and mechanical properties. Mater. Des..

[36] Zhai Y., He S.B., Lei L., Guan T.M. (2021). Mechanical property of octahedron Ti-6Al-4V fabricated by selective laser melting. Rev. Adv. Mater. Sci..

[37] Liu T., Chen D.Z., Gao X.F., Qin G., Chen R.R. (2026). Microstructure and strengthening mechanism of non-equiatomic AlCoCrFeNi high entropy alloy via Ta alloying. Intermetallics.

[38] Khrapov D., Koptyug A., Manabaev K., Léonard F., Mishurova T., Bruno G., Cheneler D., Loza K., Epple M., Surmenev R. (2020). The impact of post-manufacturing treatment of functionally graded Ti-6Al-4V scaffolds on surface morphology and mechanical strength. J. Mater. Res. Technol..

[39] Mora Sierra D.C., Heydari Astaraee A., Guagliano M., Bagherifard S. (2022). Numerical investigation of Ti-6Al-4V gradient lattice structures with tailored mechanical response. Adv. Eng. Mater..

[40] Yu G.S., Li Z.B., Li S.J., Zhang Q., Hua Y.L., Liu H., Zhao X.Y., Dhaidhai D.T., Li W., Wang X.J. (2020). The selection of internal architecture for porous Ti alloy scaffold: A compromise between mechanical properties and permeability. Mater. Des..

[41] Zhou M., Li H.H., Xiong Z.W., Li X., Li X.Y., Yang Y., Chen J., Hao S.J. (2025). NiTi alloy helical lattice structure with high reusable energy absorption and enhanced damage tolerance. J. Mater. Sci. Technol..

[42] Li J.L., Wang X.F., Guo Y., Yu X. (2019). Vertical bearing capacity of pile foundation with restriction plate via centrifuge modelling. Ocean Eng..

[43] Doan M.Q., Nguyen V.L., Le V.T., Ho D.T., Dang T.H.H., Dinh V.H., Le V.L. (2024). Enhanced energy absorption and unusual mechanical behaviors of continuously graded diamond-shellular nanostructures. Met. Mater. Int..

